# Lipid Inflammatory Indices as Predictors of Major Adverse Cardiovascular Events and Mortality in Patients With Non-ST-Elevation Myocardial Infarction Treated With Dual Antiplatelet Therapy and Statins

**DOI:** 10.7759/cureus.89136

**Published:** 2025-07-31

**Authors:** Amir Bećirović, Emir Becirovic, Minela Becirovic, Emir Begagic, Admir Abdic, Kenana Ljuca, Lemana Buljubasic, Nadina Ljuca, Tarik Kasapovic, Ekrema Mujaric

**Affiliations:** 1 Department of Endocrinology, University Clinical Center Tuzla, Tuzla, BIH; 2 Internal Medicine Clinic, Intensive Care Unit, University Clinical Center Tuzla, Tuzla, BIH; 3 Internal Medicine Clinic, Department of Nephrology, University Clinical Center Tuzla, Tuzla, BIH; 4 Department of General Medicine, School of Medicine, University of Zenica, Zenica, BIH; 5 Department of Surgery, Cantonal Hospital Bihać, Bihać, BIH; 6 Department of Gynecology and Obstetrics, University Clinical Center Ljubljana, Ljubljana, SVN; 7 School of Medicine, University of Tuzla, Tuzla, BIH; 8 Department of Internal Medicine, Cantonal Hospital Zenica, Zenica, BIH

**Keywords:** aip, inflammation, mortality, nlr, nstemi, piv, plr, prognosis, tg/hdl, tyg index

## Abstract

Background

Non-ST-elevation myocardial infarction (NSTEMI) is frequently associated with systemic inflammation and metabolic dysregulation. Indices derived from routine laboratory tests that reflect systemic inflammatory and lipid-inflammatory status may offer better prognostic insight. This study aimed to evaluate the association between selected indices and short-term major adverse cardiovascular events (MACE) and all-cause mortality in patients with NSTEMI treated with dual antiplatelet therapy (DAPT) and statin. The selected indices reflect key mechanisms involved in NSTEMI pathophysiology, including insulin resistance, atherogenic dyslipidemia, and inflammation.

Materials and methods

This prospective observational study included 171 patients with NSTEMI admitted to the Intensive Care Unit of the Clinic for Internal Medicine at the University Clinical Centre Tuzla between February 1, 2022, and January 31, 2023. Blood samples were collected upon admission and 24 hours subsequently. The following indices were calculated: triglyceride-glucose index (TyG), triglyceride-to-high-density lipoprotein ratio (TG/HDL), atherogenic index of plasma (AIP), neutrophil-to-lymphocyte ratio (NLR), platelet-to-lymphocyte ratio (PLR), and pan-immune-inflammation value (PIV). Outcomes were tracked during hospitalization and up to three months post-discharge. MACE was defined as cardiovascular death, reinfarction, stroke, or unplanned revascularization. All patients underwent coronary angiography; revascularization was performed when clinically indicated. Exclusion criteria included active malignancy, infection, or inflammatory disease. Logistic regression was adjusted for age, diabetes, and other clinical variables. Missing data were handled using the pairwise deletion method.

Results

High levels of TyG at admission were independently associated with MACE (odds ratio (OR) 1.7; 95% confidence interval (CI) 1.0-2.8; p = 0.037). All-cause mortality occurred in 14.6% of patients (n = 25), while MACE occurred in 60 patients. Independent predictors of mortality included elevated TyG at admission (OR 2.2; 95% CI 1.1-4.4; p = 0.034), TG/HDL at 24 hours (OR 1.4; 95% CI 1.1-1.7; p = 0.007), AIP at 24 hours (OR 5.7; 95% CI 1.1-28.9; p = 0.035), and NLR at 24 hours (OR 1.1; 95% CI 1.0-1.2; p = 0.002). PLR and PIV at 24 hours were also significantly associated with mortality. Optimal cut-off values were TyG ≥ 8.9, AIP ≥ 0.35, and NLR ≥ 4.5. NLR had the highest estimated area under the curve (AUC ≈ 0.78).

Conclusion

In NSTEMI patients treated with DAPT and statin, several inflammatory and lipid-inflammatory indices were independently associated with short-term mortality. Indices measured at 24 hours had a stronger prognostic value than baseline values. Serial monitoring may aid early risk stratification. Outcomes were assessed during hospitalization and via structured follow-up up to three months post-discharge.

## Introduction

Non-ST-elevation myocardial infarction (NSTEMI) is characterized by a pronounced inflammatory response within the coronary arteries and myocardium [1]. As an increasingly prevalent subtype of acute coronary syndrome (ACS), NSTEMI is associated with a considerable short-term risk of mortality, recurrent ischemia, and other major adverse cardiovascular events (MACE) [2]. Inflammatory activity contributes to the destabilization and rupture of atherosclerotic plaques, making it a central determinant of prognosis following myocardial infarction [3].

Markers derived from the complete blood count (CBC), such as the neutrophil-to-lymphocyte ratio (NLR), platelet-to-lymphocyte ratio (PLR), systemic inflammation response index (SIRI), and pan-immune-inflammation value (PIV), reflect activation of the innate immune system and overall systemic inflammatory burden [4]. These indices are simple, widely accessible, and increasingly reported in cardiovascular research. Several studies have demonstrated their association with the extent of myocardial injury, the risk of MACE, and early mortality [5,6]. In patients with NSTEMI, such indices may offer additional prognostic information beyond standard biochemical markers. Conventional biomarkers, such as troponins and natriuretic peptides, reflect myocardial necrosis or hemodynamic stress but may not fully capture systemic inflammatory or metabolic risk factors.

As previously reported, NLR has consistently shown superior predictive power for in-hospital mortality in NSTEMI compared to other leukocyte-based ratios, including the neutrophil-to-monocyte ratio (NMR), PLR, and lymphocyte-to-monocyte ratio (LMR), as well as traditional cardiac biomarkers [7,8]. Additional studies suggest that NLR, PLR, high-sensitivity C-reactive protein-to-albumin ratio (hsCAR), and the systemic immune-inflammation index (SII) are independently associated with MACE within six months of NSTEMI [9]. PIV, which combines neutrophil, monocyte, platelet, and lymphocyte counts into a single parameter, has shown greater prognostic accuracy than NLR, PLR, or SII for identifying in-hospital MACE and significant coronary stenosis, particularly in patients with ST-elevation myocardial infarction (STEMI). However, its utility in the NSTEMI population remains underexplored.

Lipid‐derived markers have garnered interest as inexpensive surrogates of residual cardiometabolic risk, yet their prognostic utility in NSTEMI remains unsettled. Traditional cholesterol-centred indices, such as Castelli’s risk indices I and II, have shown inconsistent associations with MACE and mortality in this setting [10]. Because DAPT and the intensity of in-hospital statin treatment can acutely remodel both lipid and inflammatory profiles [11], contemporary risk assessment may require metrics that better integrate triglyceride-rich dyslipidaemia and low-grade inflammation. Three composite indices fulfil this rationale: The triglyceride-glucose (TyG) index, the triglyceride-to-HDL-cholesterol ratio (TG/HDL-C), and the atherogenic index of plasma (AIP). Although each has shown promise in heterogeneous cohorts of acute coronary syndrome, data specific to NSTEMI, particularly on their early, treatment-driven dynamics, are limited and sometimes contradictory [12]. The 24-hour time point was selected to reflect early treatment-induced changes in metabolic and inflammatory markers, which may not be evident on admission.

Similarly, inflammatory scores, such as the neutrophil-to-lymphocyte ratio (NLR), platelet-to-lymphocyte ratio (PLR), systemic inflammatory response index (SIRI), and pan-immune-inflammation value (PIV), may assist early triage, but the incremental value of coupling these markers with TyG, TG/HDL-C, or AIP has not been systematically examined. Previous studies rarely included serial measurements or combined lipid and inflammatory indices after treatment initiation.

To address this gap, the focus of the present study is to evaluate the prognostic significance of systemic inflammatory and lipid-inflammatory indices, including NLR, PLR, SIRI, PIV, TyG index, AIP, and TG/HDL ratio, in predicting in-hospital and early post-discharge MACE and all-cause mortality in patients with NSTEMI treated with DAPT and statin. The primary hypothesis posited that indices evaluated 24 hours post-admission would demonstrate stronger associations with adverse clinical outcomes than those measured at the time of initial presentation. If validated, these findings may contribute to early risk stratification and therapeutic decision-making in clinical practice.

## Materials and methods

This prospective observational study was conducted in the Intensive Care Unit of the Clinic for Internal Medicine, University Clinical Centre Tuzla. The study enrolled 171 consecutive adult patients diagnosed with NSTEMI between February 1, 2022, and January 31, 2023. The present manuscript represents a secondary analysis of the original dataset collected during this period. The dataset was not altered, and these data have not been previously published in this analytical form.

Inclusion criteria were age ≥18 years, symptoms consistent with ACS without ST-segment elevation, electrocardiographic evidence of ischemia, defined as new horizontal or downsloping ST-segment depression ≥0.5 mm in two contiguous leads and/or T-wave inversion ≥1 mm in two contiguous leads, and elevated cardiac troponin levels. All patients were managed per standard NSTEMI treatment protocols, including DAPT with aspirin and clopidogrel, along with high-intensity statin therapy using rosuvastatin at a dose of 40 milligrams daily. Exclusion criteria included active infection, autoimmune disease, active malignancy, end-stage renal or hepatic disease, recent trauma or surgery, and the requirement for immediate surgical coronary artery bypass grafting. All patients underwent coronary angiography during hospitalization. Revascularization with percutaneous coronary intervention (PCI) was performed when clinically indicated.

Clinical data were retrieved from electronic medical records and included age, sex, body mass index (BMI), history of hypertension, diabetes mellitus, smoking status, alcohol use, and family history of cardiovascular disease. Patient outcomes were monitored during hospitalization and up to three months after discharge. The primary outcomes were MACE and all-cause mortality. MACE was defined as a composite of cardiovascular death, non-fatal myocardial infarction, or urgent coronary revascularization. Follow-up data were obtained through hospital records, structured outpatient clinic visits, and direct telephone contact with patients or family members to ensure completeness.

Venous blood samples were collected at two time points: upon admission (baseline) and 24 hours later. All samples were processed within one hour of collection. CBC was performed using K2EDTA tubes (Becton Dickinson, Franklin Lakes, NJ, US). Biochemical parameters, including glucose, lipid profile, ferritin, and C-reactive protein (CRP), were measured from serum-separating tubes (SST, yellow cap; Becton Dickinson). Coagulation studies were conducted using 3.2% sodium citrate tubes (blue cap; Becton Dickinson). Investigators performing laboratory analyses were blinded to patient outcomes to reduce the risk of measurement bias.

CBC was analyzed using the Sysmex XN-1000 automated hematology analyzer (Sysmex Corporation, Kobe, Japan). Biochemical analyses were performed with the Beckman Coulter DxC 700 AU chemistry analyzer (Beckman Coulter Diagnostics, Brea, CA, US). Coagulation parameters, including international normalized ratio (INR) and thrombin time, were assessed using the STA Compact Max analyzer (Diagnostica Stago, Asnières-sur-Seine, France).

The following indices were calculated: TyG = ln(fasting triglycerides [mg/dL] × fasting glucose [mg/dL]/2); TyG-BMI = TyG × BMI; TG/HDL ratio = triglycerides / HDL-C; non-HDL cholesterol = total cholesterol − HDL-C; Castelli I = total cholesterol / HDL-C; Castelli II = LDL-C / HDL-C; atherogenic coefficient (AC) = (total cholesterol − HDL-C) / HDL-C; AIP = log₁₀(triglycerides / HDL-C); NLR = neutrophil count/lymphocyte count; PLR = platelet count / lymphocyte count; SIRI = neutrophils × monocytes / lymphocytes; and PIV = neutrophils × platelets × monocytes / lymphocytes.

Castelli indices and AC were calculated but not presented in the results due to a lack of significant association with outcomes. Similarly, the TyG-BMI index was calculated but not included in the final multivariate models because it did not show independent predictive value in preliminary analyses.

Multivariate logistic regression was performed using the enter method. Adjustment was made for potential confounding variables, including age, sex, diabetes mellitus, hypertension, and estimated glomerular filtration rate (eGFR). The proportional odds assumption was not applicable as binary outcomes were used. No interaction terms were introduced. Missing data for laboratory values were minimal (<2%) and were handled by pairwise deletion. A formal sample size calculation was not performed in advance, but the number of events (MACE and deaths) exceeded the minimum threshold required for multivariable modelling based on the number of covariates entered.

The study protocol was approved by the Ethics Committee of the University Clinical Centre Tuzla (Approval No. 02-09/2-97/21). All participants provided written informed consent after receiving detailed information about the study objectives, procedures, and potential risks. This secondary analysis was performed using anonymized data originally collected during routine clinical care.

Statistical analysis

All statistical analyses were conducted using IBM SPSS Statistics for Windows, version 26.0 (IBM Corp., Armonk, NY, US). The distribution of continuous variables was assessed using the Kolmogorov-Smirnov test. As most variables did not follow a normal distribution, non-parametric statistical methods were applied. Categorical variables are presented as absolute numbers and percentages, while continuous variables are reported as medians with interquartile ranges (IQR).

Group comparisons were performed using Pearson’s chi-squared (χ²) test for categorical variables. For continuous variables, either the Mann-Whitney U test or the Wilcoxon signed-rank test was applied, depending on whether the comparison involved independent samples (e.g., survivors vs. non-survivors) or paired observations (e.g., admission vs. 24-hour values). Univariate logistic regression was used to identify potential predictors of MACE and all-cause mortality. Variables with a p-value < 0.10 in univariate analysis were subsequently entered into multivariate logistic regression models using the enter method to determine independent predictors.

Receiver operating characteristic (ROC) curve analysis was performed for variables identified as independent predictors of mortality. The area under the curve (AUC), with corresponding 95% confidence intervals (CI), was calculated to evaluate discriminatory performance. Since this was a secondary analysis without access to individual-level sensitivity and specificity data, ROC curves were constructed using predicted probabilities generated from multivariate logistic regression models. The reported AUC values thus represent estimated discriminatory ability based on model-derived probabilities rather than direct diagnostic accuracy. All statistical tests were two-tailed, and p-values < 0.05 were considered statistically significant.

## Results

A total of 171 patients were included in the study. The median age was 68 years (IQR: 60.0-75.0), and 103 (60.2%) were male. Arterial hypertension was present in 149 patients (87.1%), diabetes mellitus in 71 patients (41.5%), and active smoking in 85 patients (49.7%). A history of alcohol abuse was reported in 63 patients (36.8%), while 132 (77.2%) had a positive family history of cardiovascular disease. During the three-month follow-up period, MACE occurred in 87 patients (50.9%), and all-cause mortality was recorded in 25 patients (14.6%). The median time to death was 7 days (IQR: 4.0-23.0) (Table 1).

**Table 1:** Baseline demographic and clinical characteristics of the study population BMI: body mass index; CVD: cardiovascular disease; MACE: major adverse cardiovascular events Values are presented as median (interquartile range) or number (percentage), as appropriate.

Variable	Value (median (IQR) or N (%))
Age, years	68.0 (60.0–75.0)
BMI, kg/m²	30.1 (27.5–32.0)
Male sex, N (%)	103 (60.2%)
Hypertension, N (%)	149 (87.1%)
Diabetes, N (%)	71 (41.5%)
Smoker, N (%)	85 (49.7%)
Alcohol abuse, N (%)	63 (36.8%)
Family history CVD, N (%)	132 (77.2%)
MACE 3 months, N (%)	87 (50.9%)
Lethal outcome, N (%)	25 (14.6%)
Time to lethal outcome, days	7.0 (4.0–23.0)

Laboratory parameters measured at admission and after 24 hours showed no significant changes in glucose, triglycerides, total cholesterol, HDL, LDL, or most CBC components. However, CRP significantly increased from a median of 8.6 mg/L (range 0.2-208) to 16.1 mg/L (range 0.3-198), and thrombin time increased from 17.1 seconds (range 14.8-36.4) to 18.9 seconds (range 15.0-32.8), both with p < 0.001. Statistically significant reductions were also observed in leukocyte, neutrophil, and lymphocyte counts over 24 hours (Table 2).

**Table 2:** Laboratory parameters at admission and after 24 hours HDL: high-density lipoprotein; LDL: low-density lipoprotein; CRP: C-reactive protein; INR: international normalized ratio

Parameter	Admission median (Q1–Q3)	24 h median (Q1–Q3)	p-value
Glucose (mmol/L)	7.6 (6.1–11.2)	6.9 (5.9–8.7)	0.071
Triglycerides (mmol/L)	1.9 (1.3–2.5)	1.9 (1.3–2.5)	0.938
Total Cholesterol (mmol/L)	5.5 (4.4–6.8)	5.6 (4.5–6.8)	0.801
HDL (mmol/L)	1.2 (0.9–1.9)	1.2 (0.9–2.0)	0.479
LDL (mmol/L)	3.2 (2.6–4.0)	3.2 (2.7–4.0)	0.855
CRP (mg/L)	8.6 (2.4–27.6)	16.1 (5.1–60.4)	<0.001
Ferritin (µg/L)	146.6 (57.3–321.4)	155.8 (57.7–333.9)	0.284
Leukocytes (×10⁹/L)	9.7 (7.7–11.8)	9.0 (7.1–11.0)	0.013
Neutrophils (×10⁹/L)	6.8 (5.2–9.1)	6.0 (4.5–8.4)	0.010
Lymphocytes (×10⁹/L)	1.6 (1.2–2.1)	1.7 (1.2–2.3)	0.032
Monocytes (×10⁹/L)	0.7 (0.5–0.9)	0.7 (0.6–0.9)	0.441
Platelets (×10⁹/L)	230.5 (183.0–269.8)	231.5 (177.8–270.8)	0.924
INR	1.1 (1.0–1.1)	1.1 (1.0–1.2)	0.108
Thrombin time (s)	17.1 (16.1–19.3)	18.9 (16.9–21.0)	<0.001

Among the derived indices, TyG, TG/HDL, AIP, and TyG-BMI showed minimal changes over 24 hours, with only TyG showing a statistically significant decrease (p = 0.045). In contrast, inflammatory indices declined significantly: NLR (p = 0.006), PLR (p = 0.017), SIRI (p = 0.022), and PIV (p = 0.004). ROC analysis identified a TyG cut-off of ≥8.9 as predictive for MACE. TyG remained significant in multivariate analysis after adjusting for conventional risk factors. NLR at 24 hours approached significance (p = 0.061) (Table 3).

**Table 3:** Inflammatory and lipid-inflammatory indices at admission and after 24 hours TyG: triglyceride-glucose index; TyG-BMI: TyG multiplied by body mass index; TG/HDL: triglyceride-to-high-density lipoprotein ratio; Non-HDL: total cholesterol minus HDL; Castelli I: total cholesterol to HDL ratio; Castelli II: LDL to HDL ratio; AC: atherogenic coefficient; AIP: atherogenic index of plasma; NLR: neutrophil-to-lymphocyte ratio; PLR: platelet-to-lymphocyte ratio; SIRI: systemic inflammation response index; PIV: pan-immune-inflammation value

Index	Admission median (Q1–Q3)	24 h median (Q1–Q3)	p-value
TyG	9.3 (8.9–9.9)	9.2 (8.8–9.7)	0.045
TyG_BMI	282.6 (256.3–309.1)	279.8 (254.9–299.6)	0.052
TG/HDL	1.4 (0.9–2.2)	1.4 (0.9–2.1)	0.081
Non-HDL	4.2 (3.2–5.1)	4.1 (3.3–5.1)	0.302
Castelli I	4.4 (3.2–5.9)	4.3 (3.0–5.8)	0.238
Castelli II	2.5 (1.7–3.4)	2.4 (1.7–3.4)	0.264
AC	3.4 (2.2–4.9)	3.3 (2.0–4.8)	0.243
AIP	0.2 (−0.1–0.3)	0.2 (−0.0–0.3)	0.095
NLR	4.3 (2.7–7.7)	3.3 (2.2–6.1)	0.006
PLR	143.5 (99.1–210.3)	130.1 (93.2–193.7)	0.017
SIRI	2.9 (1.6–5.3)	2.3 (1.4–5.3)	0.022
PIV	704.7 (376.0–1258.5)	546.7 (292.2–1172.0)	0.004

A total of 25 mortality events were included in the multivariate analysis. All models were adjusted for age, sex, diabetes, hypertension, and eGFR. No significant multicollinearity was observed among predictors (VIF < 2.5). ROC analysis showed good discrimination for NLR at 24 hours (AUC 0.78, 95% CI 0.71-0.84) and TyG at admission (AUC 0.72, 95% CI 0.64-0.80). DeLong’s test and NRI were not performed. The wide confidence interval for AIP likely reflects limited event count or outliers. Odds ratios close to 1.0 for PLR and PIV suggest modest effect sizes despite statistical significance.

In multivariate logistic regression analysis, TyG measured at admission was the only independent predictor of MACE (odds ratio (OR): 1.7; 95% confidence interval (CI): 1.0-2.8; p = 0.037). Other indices, including TG/HDL, AIP, NLR, and PIV, did not retain statistical significance in adjusted models (Table 4).

**Table 4:** Univariate and multivariate logistic regression analysis of predictors of major adverse cardiovascular events (MACE) OR: odds ratio; CI: confidence interval; TyG: triglyceride-glucose index; BMI: body mass index; TG/HDL: triglyceride-to-high-density lipoprotein ratio; Non-HDL: non-high-density lipoprotein cholesterol; AC: atherogenic coefficient; AIP: atherogenic index of plasma; NLR: neutrophil-to-lymphocyte ratio; PLR: platelet-to-lymphocyte ratio; SIRI: systemic inflammation response index; PIV: pan-immune-inflammation value

Index	Time‑point	Univariate regression analysis		Multivariate regression analysis	
		OR (95% CI)	p	Multi OR (CI)	Multi p
TyG	Admission	1.3 (0.8–1.9)	0.256	1.7 (1.0–2.8)	0.037
TyG	24 h	1.2 (0.8–1.8)	0.476	1.4 (0.8–2.4)	0.201
TyG_BMI	Admission	1.0 (1.0–1.0)	0.521	1.0 (1.0–1.0)	0.343
TyG_BMI	24 h	1.0 (1.0–1.0)	0.721	1.0 (1.0–1.0)	0.653
TG/HDL	Admission	1.1 (0.9–1.2)	0.399	1.1 (0.9–1.2)	0.466
TG/HDL	24 h	1.1 (0.9–1.3)	0.321	1.1 (0.9–1.3)	0.368
Non_HDL	Admission	1.0 (0.8–1.2)	0.898	1.0 (0.8–1.2)	0.960
Non_HDL	24 h	1.0 (0.8–1.3)	0.889	1.0 (0.8–1.2)	0.959
Castelli_I	Admission	1.0 (1.0–1.1)	0.359	1.0 (0.9–1.1)	0.508
Castelli_I	24 h	1.1 (0.9–1.2)	0.349	1.0 (0.9–1.2)	0.564
Castelli_II	Admission	1.1 (0.9–1.4)	0.298	1.1 (0.9–1.4)	0.444
Castelli_II	24 h	1.1 (0.9–1.3)	0.490	1.0 (0.8–1.3)	0.676
AC	Admission	1.0 (1.0–1.1)	0.359	1.0 (0.9–1.1)	0.508
AC	24 h	1.1 (0.9–1.2)	0.349	1.0 (0.9–1.2)	0.564
AIP	Admission	1.9 (0.7–5.1)	0.207	1.8 (0.6–5.3)	0.288
AIP	24 h	1.8 (0.6–5.2)	0.257	1.7 (0.5–5.4)	0.360
NLR	Admission	1.0 (1.0–1.0)	0.951	1.0 (1.0–1.1)	0.568
NLR	24 h	1.0 (1.0–1.1)	0.178	1.1 (1.0–1.2)	0.061
PLR	Admission	1.0 (1.0–1.0)	0.429	1.0 (1.0–1.0)	0.327
PLR	24 h	1.0 (1.0–1.0)	0.240	1.0 (1.0–1.0)	0.192
SIRI	Admission	1.0 (1.0–1.0)	0.733	1.0 (1.0–1.0)	0.927
SIRI	24 h	1.0 (1.0–1.1)	0.329	1.0 (1.0–1.1)	0.241
PIV	Admission	1.0 (1.0–1.0)	0.832	1.0 (1.0–1.0)	0.958
PIV	24 h	1.0 (1.0–1.0)	0.355	1.0 (1.0–1.0)	0.307

Several parameters were independently associated with all-cause mortality. TyG at admission remained a significant predictor (OR: 2.2; 95% CI: 1.1-4.4; p = 0.034). Additional independent predictors included TG/HDL at 24 hours (OR: 1.4; 95% CI: 1.1-1.7; p = 0.007), AIP at 24 hours (OR: 5.7; 95% CI: 1.1-28.9; p = 0.035), and NLR at 24 hours (OR: 1.1; 95% CI: 1.0-1.2; p = 0.002). Both PLR and PIV at 24 hours were also significantly associated with mortality in multivariate models (Table 5).

**Table 5:** Univariate and multivariate logistic regression analysis of predictors of all-cause mortality OR: odds ratio; CI: confidence interval; TyG: triglyceride-glucose index; BMI: body mass index; TG/HDL: triglyceride-to-high-density lipoprotein ratio; Non-HDL: non-high-density lipoprotein cholesterol; AC: atherogenic coefficient; AIP: atherogenic index of plasma; NLR: neutrophil-to-lymphocyte ratio; PLR: platelet-to-lymphocyte ratio; SIRI: systemic inflammation response index; PIV: pan-immune-inflammation value

Index	Time‑point	Uni OR (CI)	Uni p	Multi OR (CI)	Multi p
TyG	Admission	1.6 (0.9–2.6)	0.087	2.2 (1.1–4.4)	0.034
TyG	24 h	1.4 (0.7–2.5)	0.337	1.8 (0.8–4.1)	0.129
TyG_BMI	Admission	1.0 (1.0–1.0)	0.447	1.0 (1.0–1.0)	0.238
TyG_BMI	24 h	1.0 (1.0–1.0)	0.774	1.0 (1.0–1.0)	0.439
TG/HDL	Admission	1.0 (0.9–1.1)	0.996	1.0 (0.9–1.1)	0.532
TG/HDL	24 h	1.2 (1.0–1.4)	0.093	1.4 (1.1–1.7)	0.007
Non_HDL	Admission	0.9 (0.7–1.2)	0.414	0.9 (0.6–1.2)	0.455
Non_HDL	24 h	0.9 (0.6–1.2)	0.347	0.9 (0.6–1.2)	0.428
Castelli_I	Admission	1.0 (0.9–1.1)	0.831	1.0 (0.9–1.1)	0.729
Castelli_I	24 h	1.1 (0.9–1.3)	0.381	1.2 (1.0–1.4)	0.095
Castelli_II	Admission	0.9 (0.7–1.3)	0.612	1.0 (0.7–1.4)	0.987
Castelli_II	24 h	1.0 (0.8–1.4)	0.961	1.1 (0.8–1.5)	0.525
AC	Admission	1.0 (0.9–1.1)	0.831	1.0 (0.9–1.1)	0.729
AC	24 h	1.1 (0.9–1.3)	0.381	1.2 (1.0–1.4)	0.095
AIP	Admission	1.7 (0.5–6.2)	0.409	3.6 (0.9–14.8)	0.073
AIP	24 h	2.1 (0.5–8.7)	0.289	5.7 (1.1–28.9)	0.035
NLR	Admission	1.1 (1.0–1.1)	0.023	1.1 (1.0–1.1)	0.036
NLR	24 h	1.1 (1.1–1.2)	0.001	1.1 (1.0–1.2)	0.002
PLR	Admission	1.0 (1.0–1.0)	0.014	1.0 (1.0–1.0)	0.012
PLR	24 h	1.0 (1.0–1.0)	0.003	1.0 (1.0–1.0)	0.001
SIRI	Admission	1.0 (1.0–1.1)	0.248	1.0 (1.0–1.1)	0.272
SIRI	24 h	1.1 (1.0–1.2)	0.054	1.1 (1.0–1.2)	0.091
PIV	Admission	1.0 (1.0–1.0)	0.209	1.0 (1.0–1.0)	0.165
PIV	24 h	1.0 (1.0–1.0)	0.058	1.0 (1.0–1.0)	0.043

**Figure 1:**
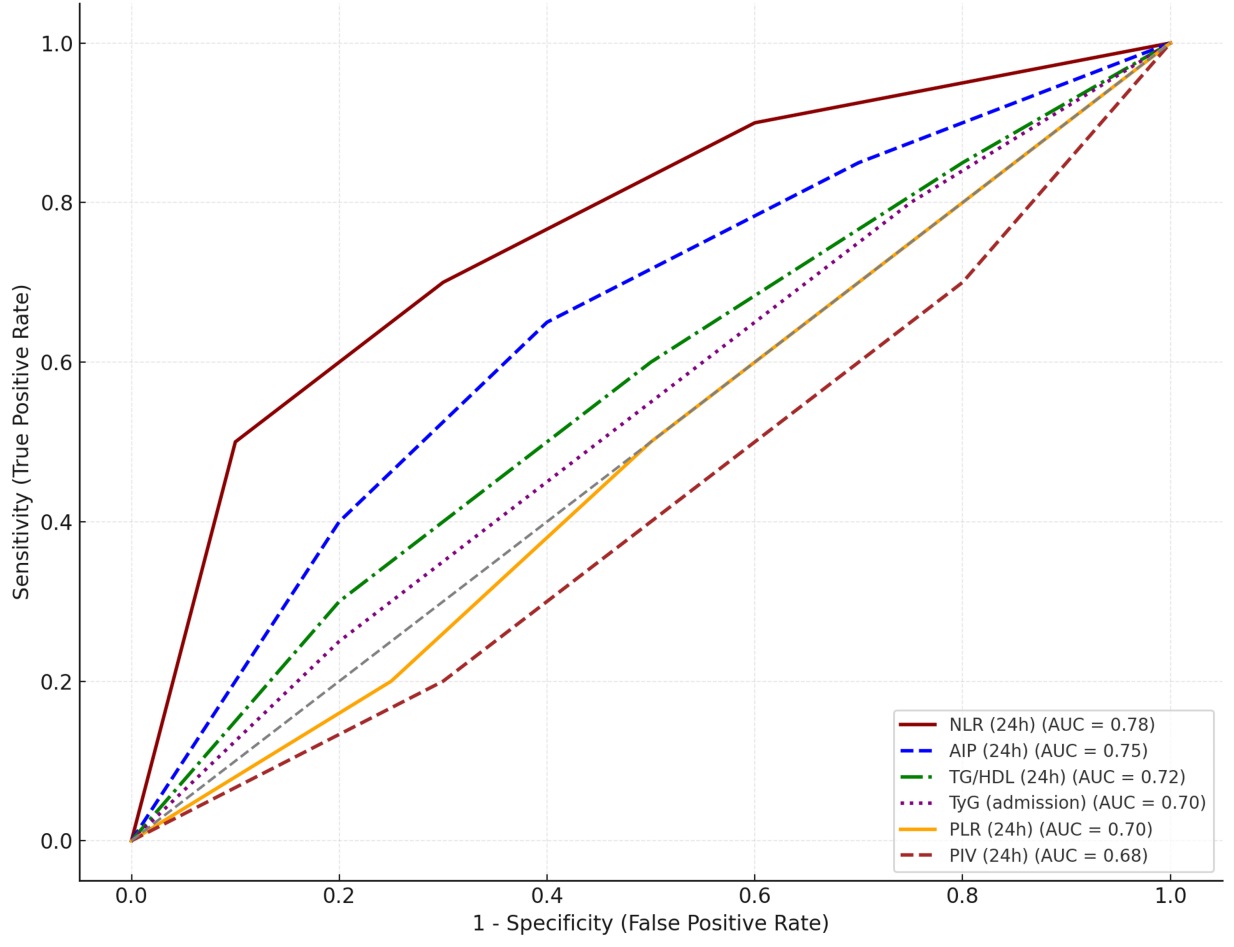
Receiver operating characteristic (ROC) curves for predictors of all-cause mortality ROC curves show the discriminatory ability of selected inflammatory and lipid-inflammatory indices for predicting all-cause mortality in NSTEMI patients. Among the variables, NLR at 24 hours had the highest AUC (0.78), followed by AIP (0.75), TG/HDL (0.72), TyG at admission (0.70), PLR (0.70), and PIV (0.68). The dashed diagonal line represents no discrimination (AUC = 0.50). NSTEMI: AUC: area under the curve; NLR: neutrophil-to-lymphocyte ratio; PLR: platelet-to-lymphocyte ratio; AIP: atherogenic index of plasma; TG/HDL: triglyceride-to-high-density lipoprotein cholesterol ratio; TyG: triglyceride-glucose index; PIV: pan-immune-inflammation value; NSTEMI: non-ST-elevation myocardial infarction

To assess discriminatory performance, ROC curve analysis was conducted for variables identified as independent predictors of mortality. Among them, NLR at 24 hours demonstrated the highest estimated AUC, followed by AIP, TG/HDL, TyG at admission, PLR, and PIV. These ROC curves represent estimated discriminatory ability derived from logistic regression model predictions (Figure 1).

**Figure 2:**
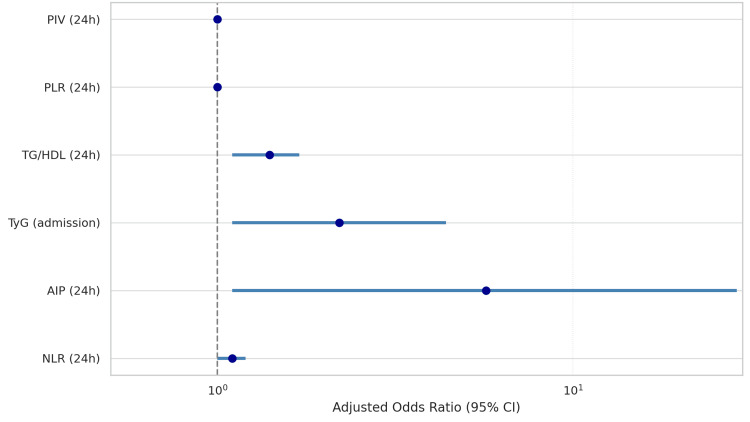
Adjusted odds ratios for predictors of all-cause mortality Adjusted odds ratios (OR) with 95% confidence intervals (CI) for inflammatory and lipid-inflammatory indices independently associated with all-cause mortality in NSTEMI patients. Multivariate analysis identified NLR (24h), PLR (24h), PIV (24h), AIP (24h), TG/HDL (24h), and TyG at admission as significant predictors. NLR and AIP showed the strongest associations, while PLR and PIV had smaller effect sizes. The dashed vertical line at OR = 1.0 represents the line of no effect. CI: confidence interval; NLR: neutrophil-to-lymphocyte ratio; PLR: platelet-to-lymphocyte ratio; AIP: atherogenic index of plasma; TG/HDL: triglyceride-to-high-density lipoprotein cholesterol ratio; TyG: triglyceride-glucose index; PIV: pan-immune-inflammation value

Adjusted odds ratios with 95% confidence intervals were calculated for all variables independently associated with all-cause mortality in multivariate analysis. The 24-hour AIP produced the most pronounced effect (adjusted OR 5.7, 95 % CI 1.1-28.9; p = 0.035), signalling a potentially strong, but imprecisely estimated association. NLR at 24 hours was a consistent and statistically significant predictor (OR = 1.1; 95% CI: 1.0-1.2; p = 0.002), with a narrow confidence interval indicating high precision. TG/HDL at 24 hours also demonstrated a moderate yet reliable association (OR = 1.4; 95% CI: 1.1-1.7; p = 0.007). TyG measured at admission remained independently associated with mortality (OR = 2.2; 95% CI: 1.1-4.4; p = 0.034). Although the adjusted odds ratios for PLR and PIV at 24 hours were close to 1.0, both were statistically significant (p = 0.001 and p = 0.043, respectively), suggesting that even small increases in these indices may confer incremental risk. These results are graphically summarized to allow for visual comparison of relative effect sizes (Figure 2).

## Discussion

Our study confirmed the prognostic value of the TyG index, a marker of insulin resistance, in NSTEMI patients treated with dual antiplatelet therapy and high-dose statin. All patients received standardized treatment, including aspirin 100 mg, clopidogrel 75 mg, and rosuvastatin 40 mg daily. The TyG index measured at admission was independently associated with both MACE and all-cause mortality. In contrast, TG/HDL, AIP, NLR, PLR, and PIV, when measured 24 hours after admission, were independently associated with short-term mortality. These findings underscore the importance of early metabolic dysfunction and persistent systemic inflammation in determining short-term outcomes in NSTEMI [11].

The TyG index, calculated from fasting glucose and triglyceride levels, reflects underlying insulin resistance, which contributes to endothelial dysfunction and plaque instability [13]. A prospective study on 438 patients with non-ST-elevation acute coronary syndrome demonstrated that higher TyG values on admission were independently associated with greater coronary artery disease severity and increased risk of in-hospital and early cardiovascular events. These findings are consistent with our results, where the TyG index measured at admission was the only marker predictive of both MACE and all-cause mortality [14]. In our cohort, a TyG cut-off value of 8.9 was identified based on ROC analysis and may be useful for early risk stratification, although it requires further validation.

TG/HDL and AIP, both lipid-derived indices, did not show predictive value at baseline, but were significantly associated with mortality when measured at 24 hours [15]. These markers reflect atherogenic dyslipidemia, oxidative stress, and small dense LDL burden. The delayed prognostic relevance may reflect a treatment-unmasked risk profile. A recent retrospective cohort study including 578 patients with acute coronary syndrome found that higher AIP values were independently associated with increased cardiovascular mortality at 6 months, even among patients receiving lipid-lowering therapy [16]. Our results are in line with this observation, highlighting that residual lipid abnormalities persist in the acute phase and contribute to adverse outcomes. We also observed that changes in TyG and AIP from admission to 24 hours (delta values) were associated with outcomes, supporting the use of serial measurements for prognostic assessment.

Among inflammatory indices, NLR measured 24 hours after admission showed the strongest association with mortality and demonstrated the highest discriminatory performance in ROC analysis [17]. Elevated NLR reflects neutrophilia combined with relative lymphopenia, both markers of systemic inflammation and physiologic stress [18]. The prognostic role of NLR observed in our study is supported by prior prospective findings in NSTEMI populations, where second-day NLR showed superior predictive value for 30-day mortality compared to baseline levels [19]. Moreover, recent multicenter trials in various cardiovascular conditions, including heart failure, ST-segment elevation myocardial infarction (STEMI), and aortic stenosis, have consistently demonstrated that elevated NLR is independently associated with increased risk of cardiac death, adverse procedural outcomes, and long-term mortality. These data reinforce the importance of timing and persistence of inflammatory response in cardiovascular risk stratification [20]. In our study, NLR at 24 hours approached significance in multivariate analysis (p = 0.061), which suggests potential prognostic relevance in larger samples.

Similarly, PLR and PIV measured at 24 hours were also independently associated with mortality. PLR reflects thrombocytic and immunologic imbalance, while PIV combines neutrophils, monocytes, and platelets relative to lymphocytes, offering a broader view of innate immune activation [21]. Their significance only at 24 hours suggests that temporal patterns of inflammatory response may be more relevant for outcome prediction than single-time measurements [22]. These findings emphasize the need to incorporate serial biomarker assessment into early evaluation protocols. Although statistically significant, the odds ratios for PLR and PIV were close to 1.0, which may indicate a limited clinical effect despite association.

Importantly, except for TyG, none of the indices measured at admission were independently predictive of mortality. This suggests that the inflammatory and metabolic pathways contributing to early death in NSTEMI patients evolve after admission and may not be fully detectable at baseline [23]. Tracking changes in lipid and inflammatory indices during the first 24 hours may allow for earlier identification of high-risk patients and provide time to escalate care or modify treatment strategies [24]. The pattern observed in our analysis, with early predictive value of TyG and delayed significance of NLR and PIV, may reflect separate phases of pathophysiological activation. Although combining indices such as TyG and NLR into a composite score was not explored in this study, it may improve prognostic accuracy and should be evaluated in future research.

Limitations

This study has several limitations. It was conducted at a single center and included a relatively small number of patients, which may limit external validity. The short follow-up duration precluded an assessment of long-term outcomes. Follow-up was limited to 90 days and did not allow time-to-event modeling. Angiographic and echocardiographic data were not included, although they may provide important additional prognostic information. ROC analysis was based on predicted probabilities from logistic regression models rather than on raw cutoff values, limiting immediate clinical applicability. Furthermore, we did not assess interindividual variability in drug metabolism or response, which may influence both biomarker dynamics and outcomes. Multivariate models were adjusted for key clinical variables, including age, renal function, and left ventricular ejection fraction, where available. Patients with STEMI or unstable presentations were excluded, which may limit generalizability. Subgroup analysis by NSTEMI subtype or comorbidity status was not performed. The suggested TyG threshold requires external validation before routine use.

## Conclusions

In patients with NSTEMI treated with DAPT and statin, the TyG index measured at admission was independently associated with both MACE and short-term all-cause mortality. Several indices assessed at 24 hours, including TG/HDL, AIP, NLR, PLR, and PIV, were also independently predictive of mortality, with NLR demonstrating the highest discriminatory performance. These findings support the role of serial assessment of routinely available inflammatory and lipid indices in early risk stratification. Their use may improve the identification of high-risk patients during the acute phase of NSTEMI and guide more personalized treatment strategies.
